# Transcriptomics-guided serum podoplanin and interpretable machine learning for early risk stratification of necrotizing enterocolitis in preterm infants

**DOI:** 10.3389/fimmu.2026.1973760

**Published:** 2026-10-06

**Authors:** Hanghang Peng, Xue Liu, Yihui Li, Minqian Zhou, Jingtao Bian, Yuhan Huang, Zengxue Hu, Wenqiang Sun, Xueping Zhu

**Affiliations:** 1 Department of Neonatology, Children’s Hospital of Soochow University, Suzhou, China; 2 Suzhou Medical College, Soochow University, Suzhou, China

**Keywords:** biomarkers, machine learning, necrotizing enterocolitis, podoplanin, preterm infants, transcriptomics

## Abstract

**Background:**

To evaluate podoplanin (PDPN) as a circulating biomarker for early necrotizing enterocolitis (NEC) risk stratification and to determine whether an interpretable machine-learning framework integrating PDPN with prediagnostic clinical and inflammatory variables could improve individualized risk estimation.

**Methods:**

PDPN expression was examined in two public NEC intestinal transcriptomic datasets. Serum PDPN was then measured by enzyme-linked immunosorbent assay in a nested case-control cohort of 132 preterm infants (66 with NEC and 66 gestational-age- and birth-weight-matched controls). All candidate predictors were obtained before NEC onset in cases and before the matched reference time in controls. Restricted cubic spline, receiver operating characteristic, precision-recall, LASSO, multivariable logistic regression, 11 machine-learning algorithms, SHAP, and nomogram analyses were performed.

**Results:**

PDPN was upregulated in NEC intestinal tissue. Serum concentrations were higher in infants who developed NEC than in controls (48.36 ± 22.89 vs 19.75 ± 6.26 ng/mL, P < 0.001) and in Bell stage III than in Bell stage II NEC [69.43 (58.91–77.95) vs 30.59 (24.06–42.79) ng/mL, P < 0.001]. PDPN correlated weakly with the C-reactive protein-to-albumin ratio and high-sensitivity C-reactive protein, achieved an area under the receiver operating characteristic curve of 0.905 and an area under the precision-recall curve of 0.925, and remained independently associated with NEC after multivariable adjustment. CatBoost showed the most favorable internal performance among 11 models, with PDPN as the leading SHAP contributor. The nomogram achieved an internally assessed area under the curve of 0.974.

**Conclusions:**

Prediagnostic serum PDPN, particularly when combined with routinely available variables, may support early individualized NEC risk stratification. Prospective multicenter validation and mechanistic studies are warranted.

## Introduction

Necrotizing enterocolitis (NEC) is among the most serious gastrointestinal emergencies affecting preterm infants, especially those born at very low gestational ages with immature intestinal function. Its pathology involves intestinal inflammation, loss of epithelial barrier integrity, microbial dysregulation, impaired perfusion, and progressive tissue injury (1–3). Early risk stratification remains difficult despite advances in neonatal intensive care. Initial manifestations are often nonspecific and overlap with feeding intolerance, infection, or other neonatal complications; by the time characteristic radiographic or systemic signs appear, intestinal injury may already be advanced (4–6). Biomarkers that are clinically accessible and can identify vulnerable infants before overt deterioration are therefore needed.

NEC arises through interacting disturbances in the intestinal epithelium, innate immune response, vascular and lymphatic function, platelet signaling, and systemic inflammation (7, 8). Blood cell counts, C-reactive protein, procalcitonin, and derived immunoinflammatory indices are readily available and may capture part of the inflammatory milieu preceding disease onset. Their interpretation is difficult, however, because gestational age, infection, transfusion, antibiotic exposure, and normal postnatal adaptation can alter these measures (9). No routine inflammatory marker alone has proved sufficiently reliable for early NEC risk stratification in clinical practice (6, 10–12).

Podoplanin (PDPN) is a mucin-type transmembrane glycoprotein with roles in inflammatory activation, lymphatic remodeling, platelet-CLEC-2 signaling, immune-cell trafficking, and epithelial repair (13–16). These functions intersect with several features of NEC, notably intestinal inflammation, microcirculatory disturbance, and mucosal injury (7, 13). Although PDPN has been studied in inflammatory and vascular disorders, its potential as a circulating biomarker for NEC in preterm infants remains uncertain (17, 18). On this biological basis, we hypothesized that circulating PDPN could help identify preterm infants at risk for NEC before clinical diagnosis.

Machine-learning methods offer a way to combine clinical, laboratory, and biomarker data while accommodating nonlinear relationships in neonatal risk prediction (19). Their clinical value nevertheless depends on adequate validation, biological coherence, and outputs that clinicians can interpret. SHapley Additive exPlanations (SHAP) can show how each predictor influences model output at the cohort and individual levels (20). Guided by this hypothesis, we first assessed PDPN expression in two public NEC intestinal-tissue datasets and then measured serum PDPN before diagnosis in a matched clinical cohort. Clinical, laboratory, and immunoinflammatory predictors were subsequently integrated using conventional statistics, machine learning, SHAP interpretation, and a nomogram to evaluate early NEC risk.

## Materials and methods

### Study design and participants

This nested case-control study was conducted in the Department of Neonatology at the Children’s Hospital of Soochow University, Suzhou, China. Preterm infants born at <32 weeks of gestation and admitted within 24 h of birth between January 1, 2023, and June 1, 2025, were screened. Cases were infants who developed NEC meeting the criteria for modified Bell stage II or higher (21, 22). Contemporaneous infants who remained free of NEC throughout hospitalization served as controls and were matched 1:1 to cases by gestational age (± 3 days) and birth weight (± 100 g).

Infants were excluded if they had a known chromosomal abnormality or inherited metabolic disorder, a major congenital structural malformation, or no parental or guardian consent. After eligibility assessment and matching, the study included 132 infants: 66 with NEC and 66 matched controls. NEC severity was classified using the modified Bell staging system.

This study was reviewed and approved by the Institutional Review Board of the Children’s Hospital of Soochow University (approval no. 2025CS231) and was conducted in accordance with the Declaration of Helsinki and applicable institutional and national requirements. Written informed consent to participate was obtained from the parents or legal guardians of all enrolled infants.

Serum PDPN, clinical variables, laboratory measurements, and derived immunoinflammatory biomarkers were obtained from the same cohort. Candidate predictors were limited to information available before NEC onset or clinical diagnosis, consistent with the aim of early risk stratification rather than retrospective disease classification. For each control, clinical and laboratory data were drawn, whenever possible, from the postnatal window corresponding to that of the matched case.

### Transcriptomic assessment of PDPN expression

To determine whether the prespecified candidate PDPN was differentially expressed in NEC intestinal tissue, we obtained GSE46619 and GSE64801 from the Gene Expression Omnibus (23). NEC and control intestinal samples were compared separately within each dataset.

After standard preprocessing, the microarray dataset GSE46619 was analyzed with limma (24), and the RNA-sequencing dataset GSE64801 was analyzed with DESeq2 (25). Differential expression was defined as an adjusted P value < 0.05 with an absolute log2 fold change > 1. Volcano plots summarized the genome-wide results, after which PDPN expression was examined across both datasets.

These analyses were designed to provide tissue-level evidence for the prespecified PDPN hypothesis before evaluating the biomarker in serum collected prior to clinical NEC diagnosis.

### Serum sample collection and PDPN measurement

Residual serum remaining after routine laboratory tests performed at approximately 24 h after birth, postnatal day 3, the end of the first week, and weekly thereafter was obtained from the institutional preterm-infant cohort biobank. Under standardized biobank procedures, samples were processed within 2 h of collection, divided into aliquots, assigned unique identifiers, and stored at -80 °C until use. The time of clinical diagnosis served as the index time for cases. We selected case samples obtained 3–5 days before that index time; control samples were chosen from postnatal ages corresponding to those of the matched cases, with an allowable difference of ±3 days.

Serum PDPN was quantified with a commercially available human enzyme-linked immunosorbent assay kit (ELK Biotechnology, ELK3051, Wuhan, China) following the manufacturer’s protocol. Concentrations were derived from a standard curve generated for each assay plate. Laboratory personnel were blinded to the clinical groups whenever possible.

### Definitions and clinical data collection

NEC was diagnosed and staged according to the modified Bell criteria (21, 22). Cases comprised infants with stage II or III disease; these stages were also used for severity subgroup analyses. Controls were matched preterm infants who did not develop NEC during hospitalization.

Trained investigators extracted clinical information from electronic medical records. Maternal and perinatal data covered maternal age, gestational hypertension, gestational diabetes mellitus, premature rupture of membranes, antenatal medication exposure, mode of delivery, gestational age, birth weight, sex, small-for-gestational-age status, and Apgar scores. Standard neonatal clinical criteria were used to define respiratory distress syndrome, hemodynamically significant patent ductus arteriosus, bronchopulmonary dysplasia, retinopathy of prematurity, atrial or ventricular septal defect, early-onset sepsis, and late-onset sepsis (26).

Treatment and nutritional variables were recorded up to NEC onset in cases and through the corresponding postnatal time point in controls. They comprised respiratory support, pulmonary surfactant, caffeine, red blood cell transfusion, peripherally inserted central and umbilical venous catheters, antibiotic exposure, initiation and duration of enteral feeding, and attainment of full enteral feeding. Premature rupture of membranes >24 h denoted rupture more than 24 h before delivery; time to first enteral feeding >24 h denoted feeding initiated after the first postnatal day. Antibiotic and enteral-feeding durations were calculated from birth to NEC onset or the matched reference time.

Laboratory predictors, including the complete blood count, high-sensitivity C-reactive protein, procalcitonin, albumin, and lactate dehydrogenase, were obtained within the same prespecified window as PDPN sampling. For cases, this window was 3–5 days before NEC diagnosis, and the result closest to PDPN sampling within this window was selected. Laboratory results from the corresponding postnatal-age window were used for matched controls.

### Calculation of immunoinflammatory biomarkers

Immunoinflammatory biomarkers were calculated from the routine laboratory measurements selected within this same prespecified sampling window. The complete blood count provided platelet, neutrophil, lymphocyte, and monocyte counts; albumin, lactate dehydrogenase, procalcitonin, and high-sensitivity C-reactive protein were obtained from biochemical and inflammatory testing. Blood cell counts were converted to consistent units before calculation.

The derived biomarkers were the platelet-to-lymphocyte ratio (PLR = platelet count/lymphocyte count), neutrophil-to-lymphocyte ratio (NLR = neutrophil count/lymphocyte count), systemic immune-inflammation index (SII = platelet count × neutrophil count/lymphocyte count), lymphocyte-to-monocyte ratio (LMR = lymphocyte count/monocyte count), systemic inflammation response index (SIRI = neutrophil count × monocyte count/lymphocyte count), platelet-to-neutrophil ratio (PNR = platelet count/neutrophil count), neutrophil-to-monocyte ratio (NMR = neutrophil count/monocyte count), and C-reactive protein-to-albumin ratio (CAR = high-sensitivity C-reactive protein/albumin).

Monocyte count, lactate dehydrogenase, procalcitonin, and high-sensitivity C-reactive protein were also examined as markers of inflammation or tissue injury.

### Statistical analysis

Normality of continuous variables was assessed with the Shapiro-Wilk test. Data with a normal distribution are reported as mean ± standard deviation and were compared by Student’s t-test. Non-normally distributed data are given as median and interquartile range and were compared by the Mann-Whitney U test. Categorical variables are reported as number and percentage; comparisons used the chi-square test or Fisher’s exact test, as appropriate.

Univariable logistic regression was used to examine associations between individual pre-NEC variables and NEC occurrence. The main table presents selected clinically relevant baseline characteristics, with the complete baseline profile reported in the **Supplementary Table S1**. Effect estimates are expressed as odds ratios (ORs) with 95% confidence intervals (CIs). Because cases and controls were individually matched on gestational age and birth weight, baseline comparisons and logistic estimates were interpreted in the context of this matched design.

Associations between serum PDPN and immunoinflammatory biomarkers were assessed using Spearman correlation analysis. Restricted cubic splines were fitted to examine dose-response relationships with NEC occurrence. Individual discriminative performance was evaluated using receiver operating characteristic curves and their corresponding areas under the curve. For serum PDPN, candidate cutoff values were defined as the midpoints between consecutive distinct observed concentrations, and the cutoff maximizing the Youden index was selected. In the event of a tie, the cutoff yielding higher sensitivity was preferred. Precision-recall curves and their corresponding areas under the curve were also obtained (27). All statistical tests were two-sided, with P < 0.05 considered statistically significant. Analyses were performed using SPSS and R.

### Feature selection and machine learning model development

All candidate predictors were measured or documented before NEC onset in cases and before the matched index time in controls. The cohort was randomly divided into training and testing sets in a 7:3 ratio before feature selection. Least absolute shrinkage and selection operator (LASSO) regression was performed exclusively within the training set, with the penalty parameter selected by cross-validation within that set (28). Eight predictors with non-zero coefficients were retained for subsequent modeling.

Eleven algorithms were compared: CatBoost, extreme gradient boosting, support vector machine, k-nearest neighbors, random forest, naive Bayes, gradient boosting decision tree, decision tree, adaptive boosting, logistic regression, and LightGBM (29–31). Performance was assessed in terms of discrimination, precision-recall, calibration, and clinical utility (27, 32, 33). Reported metrics included accuracy, sensitivity, specificity, precision, F1-score, Matthews correlation coefficient, area under the receiver operating characteristic curve, and area under the precision-recall curve. Model behavior was also examined using receiver operating characteristic and precision-recall curves, calibration plots, and decision curve analysis. CatBoost had the most favorable internal performance and was taken forward for interpretation.

### SHAP interpretation and nomogram construction

SHAP analysis was applied to the final CatBoost model to make its predictions more interpretable (20). Predictor contributions were quantified globally and for individual infants. Global importance was defined by the mean absolute SHAP value. Summary, beeswarm, heatmap, line, heatwave, and dependence plots displayed the rank, direction, magnitude, and between-infant variation of feature contributions.

For the final nomogram, a logistic regression model incorporating the eight predictors selected in the training set was refitted using all 132 infants. This full-sample analysis was separate from the 7:3 split used for the machine-learning analyses. Calibration was assessed using 1,000 bootstrap resamples, with the predictor set held fixed and the regression coefficients re-estimated in each resample. Calibration curves, mean absolute calibration error, and optimism-corrected calibration intercept and slope were reported. Discrimination was evaluated using receiver operating characteristic (ROC) analysis. Potential clinical utility was explored using decision curve analysis (DCA) and a clinical impact curve (CIC). Residual-versus-fitted plots were examined as model diagnostics.

## Results

### Study population and overall analytical workflow

The final cohort comprised 132 preterm infants, with 66 NEC cases and 66 matched controls. Serum PDPN and all candidate model variables came from these infants, and every predictor entered into machine learning had been recorded before NEC onset. Of the 66 cases, 39 had Bell stage II and 27 had stage III disease (**Table 1**, **Supplementary Table S1**).

**Table 1:** Selected baseline characteristics of infants with NEC and matched controls.

Variables	Control (n=66)	NEC (n=66)	*P*-value
Gestational age, weeks	30.08 (1.94)	29.72 (2.07)	0.303
Birth weight, g	1218.79 (342.94)	1229.79 (316.54)	0.848
Male sex, n (%)	37.00 (56.06%)	36.00 (54.55%)	0.861
Cesarean delivery, n (%)	34.00 (51.52%)	31.00 (46.97%)	0.601
SGA, n (%)	20.00 (30.30%)	19.00 (28.79%)	0.849
PROM >24 h, n (%)	18.00 (27.27%)	9.00 (13.64%)	0.052
Time to first enteral feeding >24 h, n (%)	5.00 (7.58%)	10.00 (15.87%)	0.142
Duration of enteral feeding before NEC, days	28.83 (12.95)	18.18 (14.17)	<0.001
Duration of antibiotic use before NEC, days	9.23 (4.68)	13.84 (9.54)	<0.001
Full enteral feeding before NEC, n (%)	25.00 (37.88%)	36.00 (57.14%)	0.028
Monocyte count, ×10^9^/L	1.75 (0.89)	1.44 (1.09)	0.072
Platelet count, ×10^9^/L	326.09 (126.87)	250.48 (137.84)	0.001
Hemoglobin, g/L	123.14 (22.51)	113.74 (26.00)	0.029
RDW, %	15.87 (1.89)	16.62 (2.26)	0.043
PCT, ng/mL	0.36 (0.12)	0.30 (0.16)	0.037
hs-CRP, mg/L	4.27 (10.37)	9.66 (17.21)	0.052
LDH, U/L	375.83 (186.76)	440.49 (211.93)	0.080
CAR	0.12 (0.31)	0.32 (0.73)	0.053
Serum PDPN, ng/mL	19.75 (6.26)	48.36 (22.89)	<0.001
PLR	76.93 (28.72)	71.01 (39.47)	0.328
NLR	1.05 (0.81)	1.15 (0.87)	0.463
SII	316.13 (246.13)	268.09 (243.24)	0.263
LMR	3.04 (1.59)	3.69 (2.36)	0.070
SIRI	2.11 (2.49)	2.02 (3.07)	0.854
PNR	110.73 (77.15)	98.25 (77.33)	0.357
NMR	2.51 (1.20)	5.22 (11.68)	0.066

Data are presented as mean ± standard deviation or median with interquartile range for continuous variables and as number with percentage for categorical variables, as appropriate. SGA, Small for gestational age; PROM, Premature rupture of membranes; NEC, necrotizing enterocolitis; RDW, Red cell distribution width; PCT, Procalcitonin; hs-CRP, High-sensitivity C-reactive protein; LDH, Lactate dehydrogenase; CAR, C-reactive protein-to-albumin ratio; PDPN, podoplanin; PLR, platelet-to-lymphocyte ratio; NLR, neutrophil-to-lymphocyte ratio; SII, systemic immune-inflammation index; LMR, lymphocyte-to-monocyte ratio; SIRI, systemic inflammation response index; PNR, platelet-to-neutrophil ratio; NMR, neutrophil-to-monocyte ratio.

The analytical workflow first examined PDPN expression in two public NEC datasets and then evaluated prediagnostic serum PDPN in the matched clinical cohort. Serum concentrations were compared between cases and controls and across Bell stages, followed by correlations with derived immunoinflammatory biomarkers. Dose-response patterns and individual biomarker performance were assessed using restricted cubic splines, receiver operating characteristic curves, and precision-recall curves. LASSO selected features for multivariable logistic regression and machine-learning models. The best-performing model was interpreted with SHAP, while a nomogram provided individualized risk estimates (**Figure 1**).

**Figure 1:**
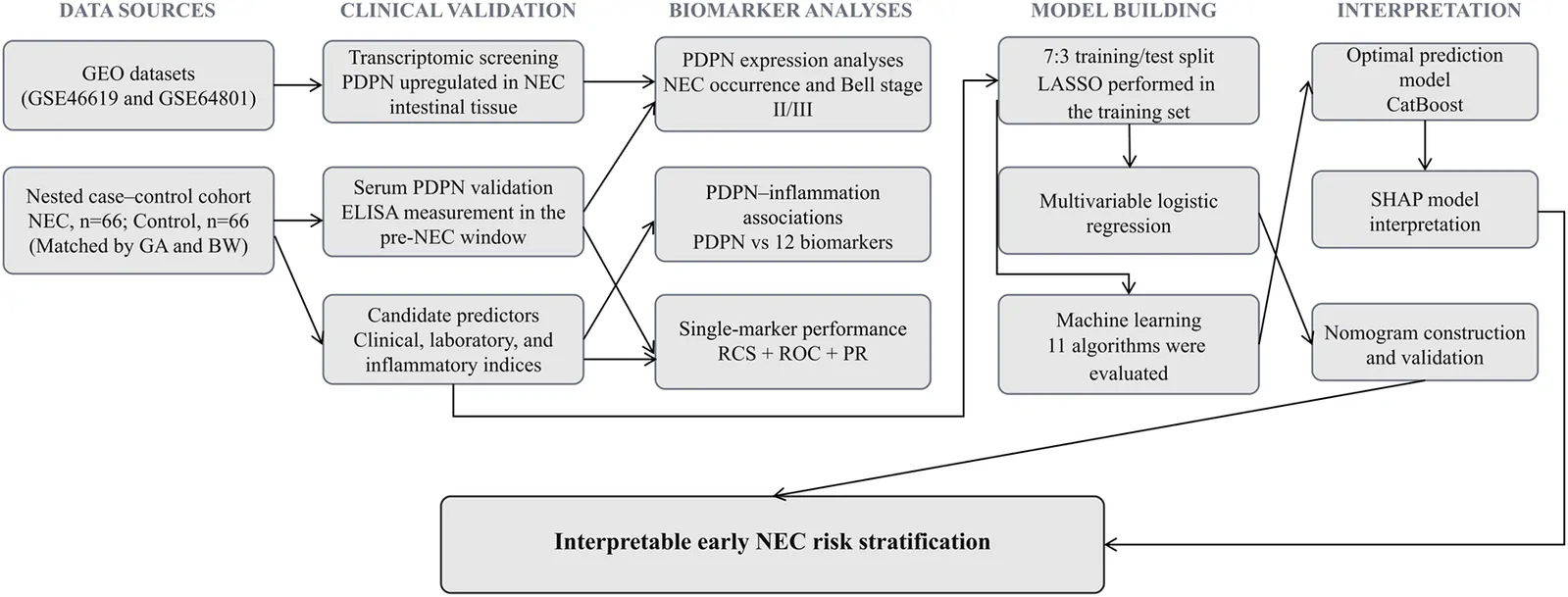
Study population and overall analytical workflow.

### Transcriptomic evidence and serum validation of PDPN

PDPN was upregulated in NEC intestinal tissue in both GSE46619 and GSE64801 (**Figures 2A, B**). The concordant tissue-level findings provided transcriptomic support for evaluating the prespecified biomarker in serum.

**Figure 2:**
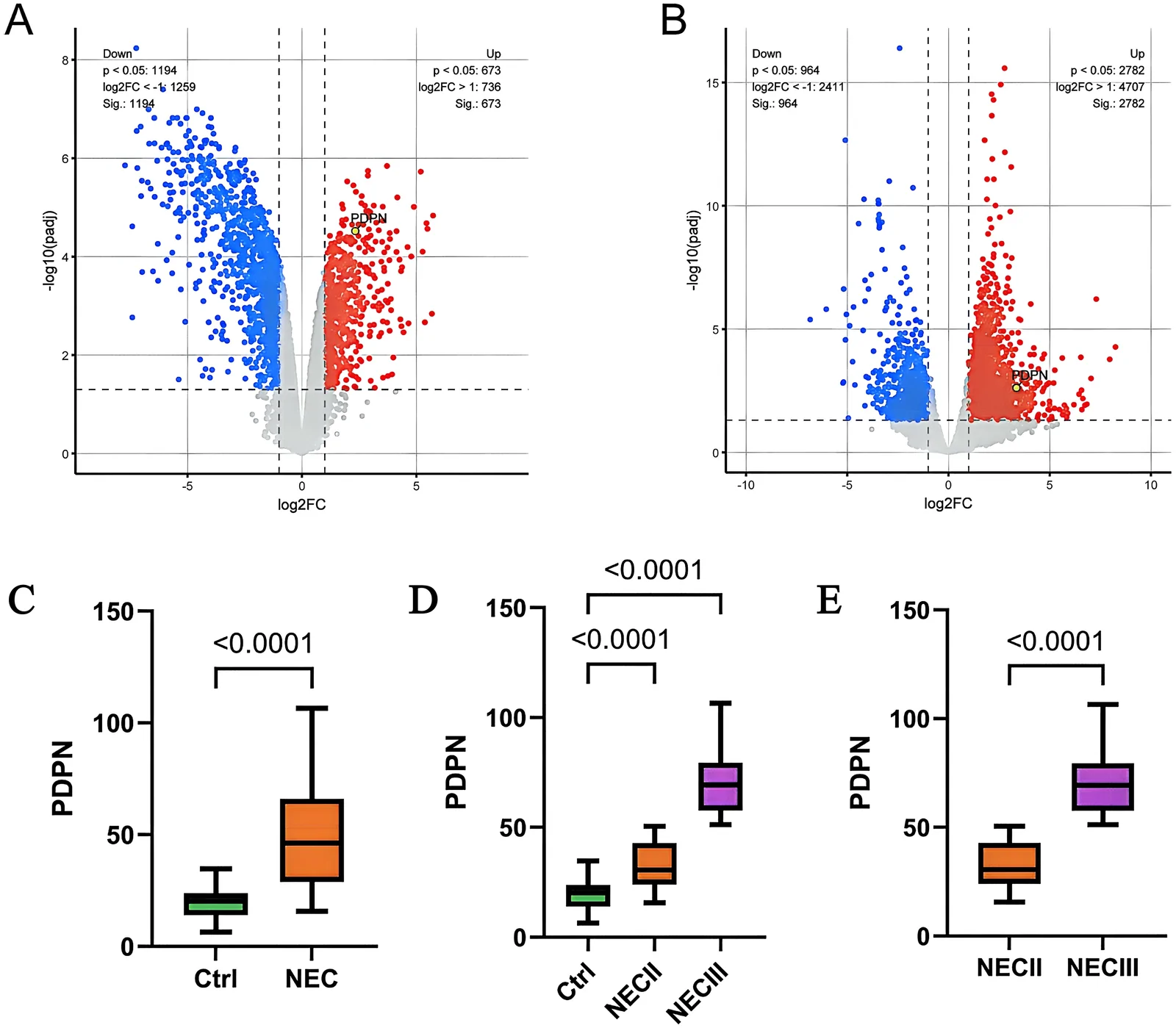
Transcriptomic evidence and serum validation of PDPN. **(A, B)** Differential expression of PDPN in NEC intestinal tissue across two public transcriptomic datasets. **(C)** Serum PDPN levels in matched controls and infants with NEC. **(D)** Serum PDPN levels across controls, Bell stage II NEC, and Bell stage III NEC. **(E)** Serum PDPN levels in infants with Bell stage II and Bell stage III NEC.

Serum PDPN was higher in infants who subsequently developed NEC than in matched controls (48.36 ± 22.89 vs. 19.75 ± 6.26 ng/mL, P < 0.001; **Figure 2C**). Concentrations rose across controls, Bell stage II NEC, and Bell stage III NEC (**Figure 2D**). Stage III cases also had markedly higher levels than stage II cases [69.43 (58.91-77.95) vs. 30.59 (24.06-42.79) ng/mL, P < 0.001; **Figure 2E**]. Thus, circulating PDPN was associated with both NEC occurrence and clinical severity.

### Serum PDPN and inflammatory biomarkers

Serum PDPN correlated weakly but significantly with CAR (Spearman R = 0.196, P = 0.037) and high-sensitivity C-reactive protein (Spearman R = 0.290, P = 0.003; **Figures 3H, L**). Correlations with PLR, NLR, SII, LMR, SIRI, PNR, NMR, monocyte count, lactate dehydrogenase, and procalcitonin were not significant (all P > 0.05; **Figures 3A–G, I–K**). PDPN may therefore reflect a component of CRP-related systemic inflammation while retaining information not captured by cell-count-derived indices.

**Figure 3:**
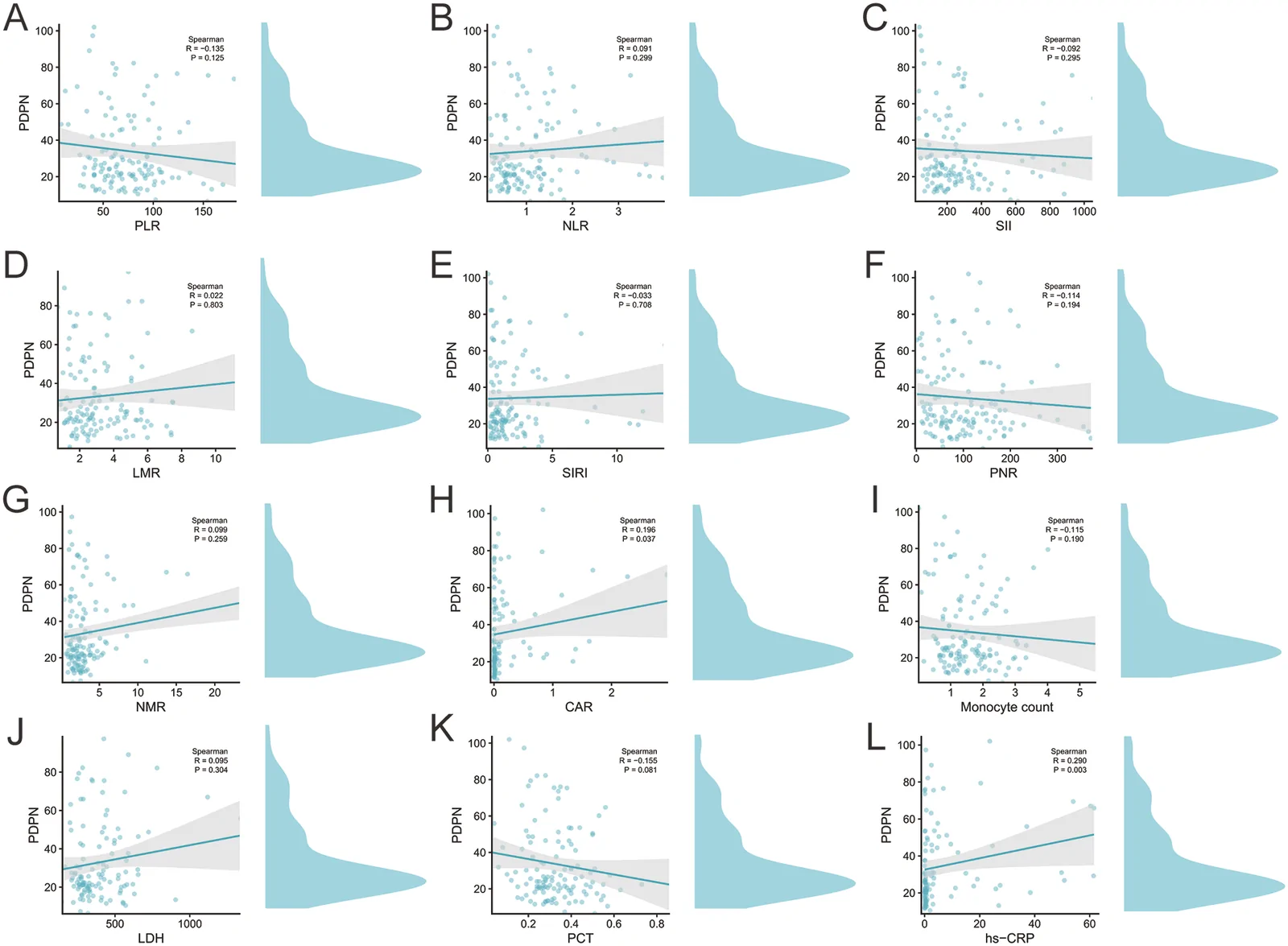
Correlations between serum PDPN and immunoinflammatory biomarkers. Spearman correlation analyses were performed to evaluate the associations between serum PDPN and PLR **(A)**, NLR **(B)**, SII **(C)**, LMR **(D)**, SIRI **(E)**, PNR **(F)**, NMR **(G)**, CAR **(H)**, monocyte count **(I)**, LDH **(J)**, PCT **(K)**, and high-sensitivity C-reactive protein **(L)**.

### Dose-response relationships and individual biomarker performance

Restricted cubic splines revealed differing dose-response patterns across PDPN and the immunoinflammatory biomarkers (**Figure 4**). The heterogeneity of these curves supported multivariable evaluation rather than reliance on any single conventional inflammatory measure.

**Figure 4:**
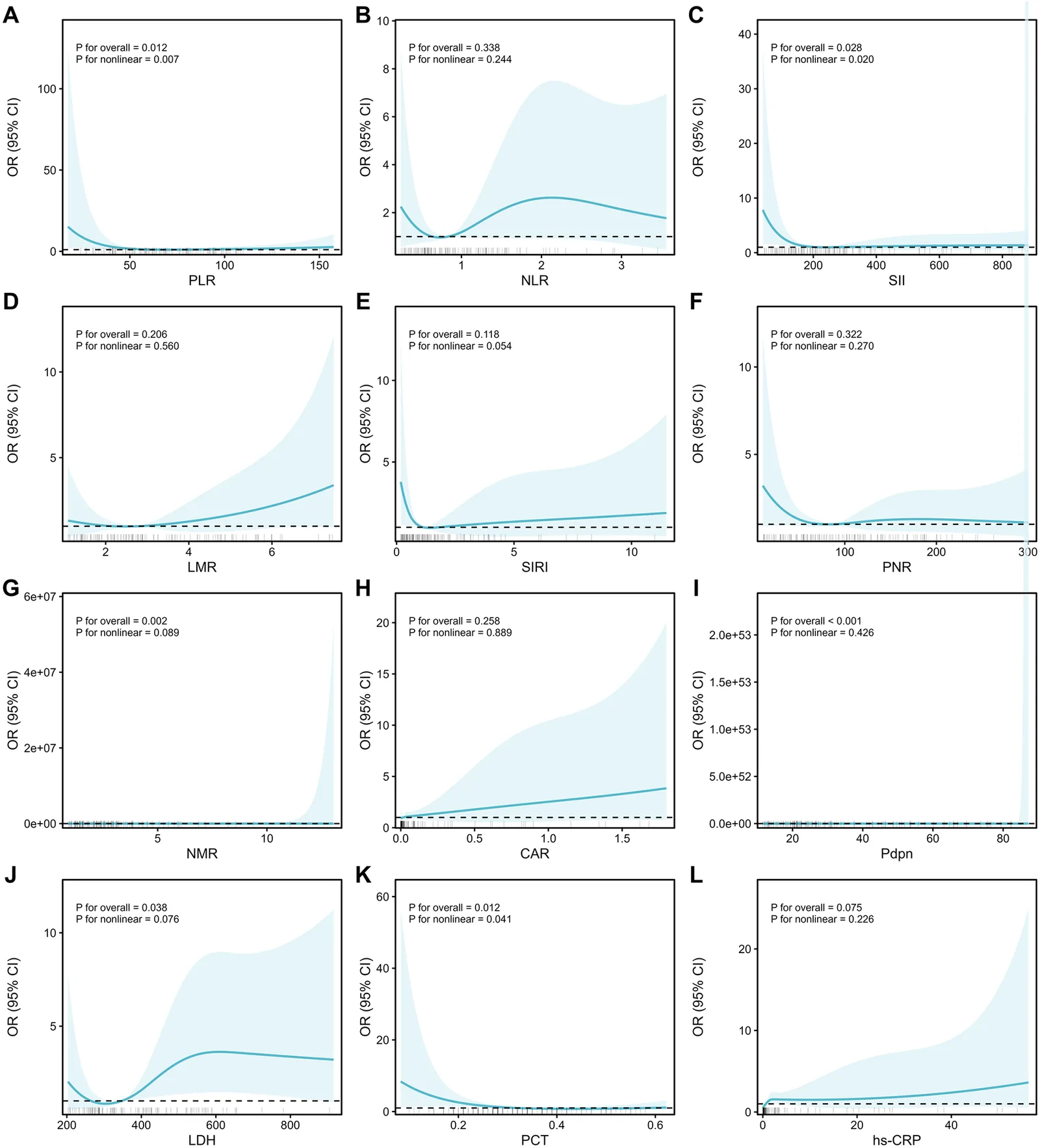
Restricted cubic spline analyses of PDPN and immunoinflammatory biomarkers for NEC risk. Restricted cubic spline models evaluated dose-response relationships between NEC risk and PLR **(A)**, NLR **(B)**, SII **(C)**, LMR **(D)**, SIRI **(E)**, PNR **(F)**, NMR **(G)**, CAR **(H)**, PDPN **(I)**, LDH **(J)**, PCT **(K)**, and high-sensitivity C-reactive protein **(L)**.

PDPN provided the strongest individual discrimination, with an area under the receiver operating characteristic curve of 0.905 and an area under the precision-recall curve of 0.925 (**Figures 5A, B**). At the Youden-derived cutoff of 27.68 ng/mL, with values ≥27.68 ng/mL classified as positive, sensitivity was 77.3% and specificity was 90.9%. Corresponding receiver operating characteristic areas for the other immunoinflammatory biomarkers were generally 0.537–0.644. Although these conventional measures performed modestly in isolation, they may still add complementary information to multivariable models.

**Figure 5:**
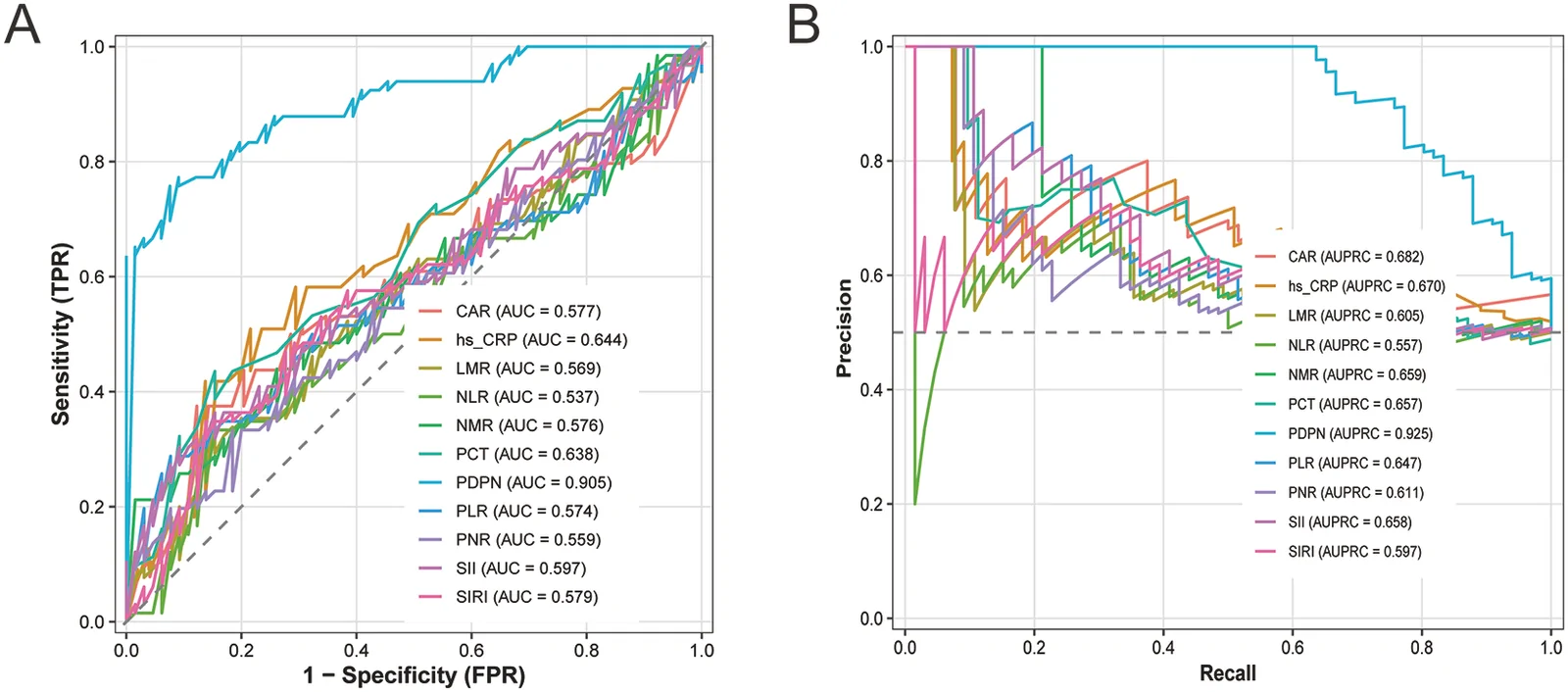
Receiver operating characteristic and precision-recall analyses of PDPN and immunoinflammatory biomarkers for NEC identification. **(A)** ROC curves comparing the individual diagnostic performance. **(B)** PR curves comparing the predictive performance.

### LASSO-based feature selection and multivariable logistic regression

LASSO analysis within the training set retained eight predictors with non-zero coefficients: serum PDPN, monocyte count, premature rupture of membranes >24 h, time to first enteral feeding >24 h, duration of enteral feeding before NEC, duration of antibiotic use before NEC, hemoglobin, and red cell distribution width (**Figures 6A, B**).

**Figure 6:**
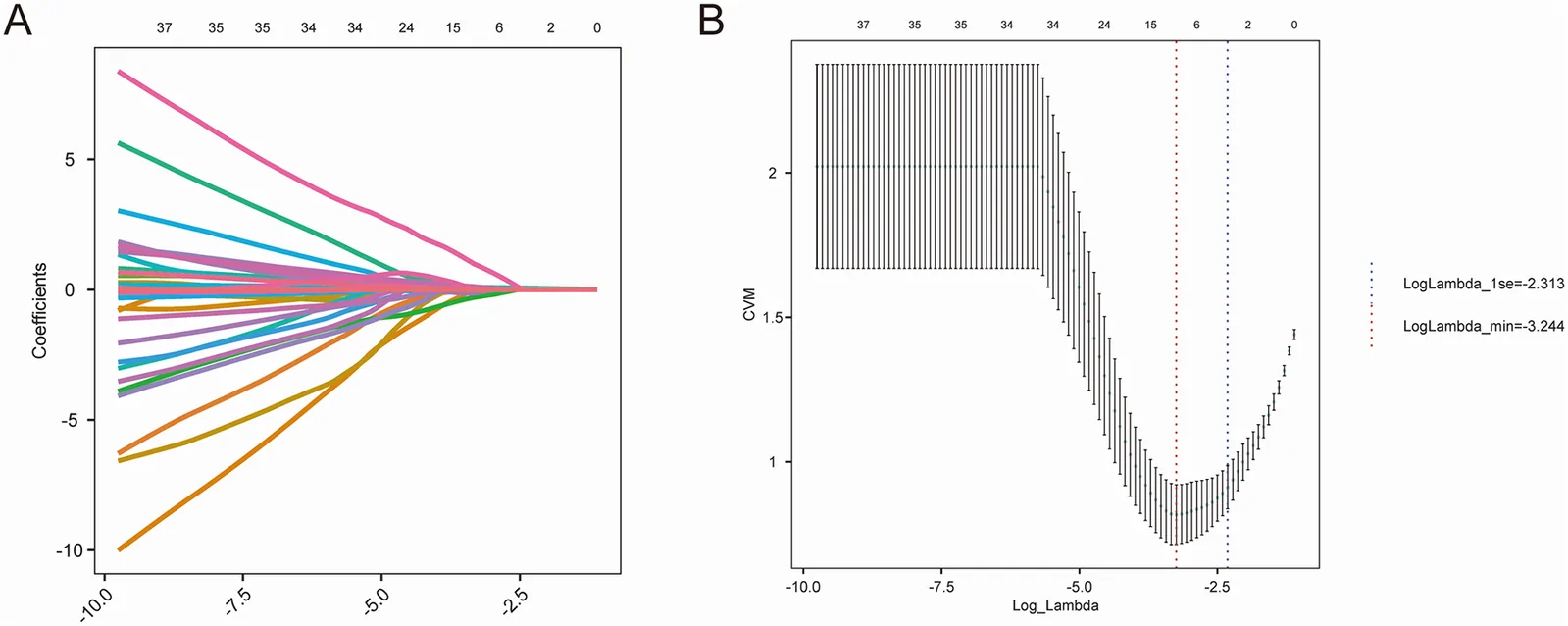
LASSO-based feature selection. **(A)** LASSO coefficient profiles. **(B)** Selection of the optimal penalty parameter (lambda) by cross-validation within the training set.

All eight predictors were entered into multivariable logistic regression (**Figure 7**). PDPN remained associated with NEC after adjustment (OR, 1.247; 95% CI, 1.141-1.414; P < 0.001). Longer pre-NEC antibiotic exposure was associated with higher odds (OR, 1.172; 95% CI, 1.066-1.328; P = 0.004), whereas a longer duration of enteral feeding was associated with lower odds (OR, 0.933; 95% CI, 0.869-0.986; P = 0.025). Initiation of enteral feeding after 24 h was also associated with NEC (OR, 11.374; 95% CI, 1.768-89.138; P = 0.013), although the wide CI denotes considerable uncertainty. Monocyte count showed an inverse association (OR, 0.354; 95% CI, 0.150-0.792; P = 0.011). Hemoglobin, red cell distribution width, and premature rupture of membranes >24 h were not significant in the adjusted model.

**Figure 7:**
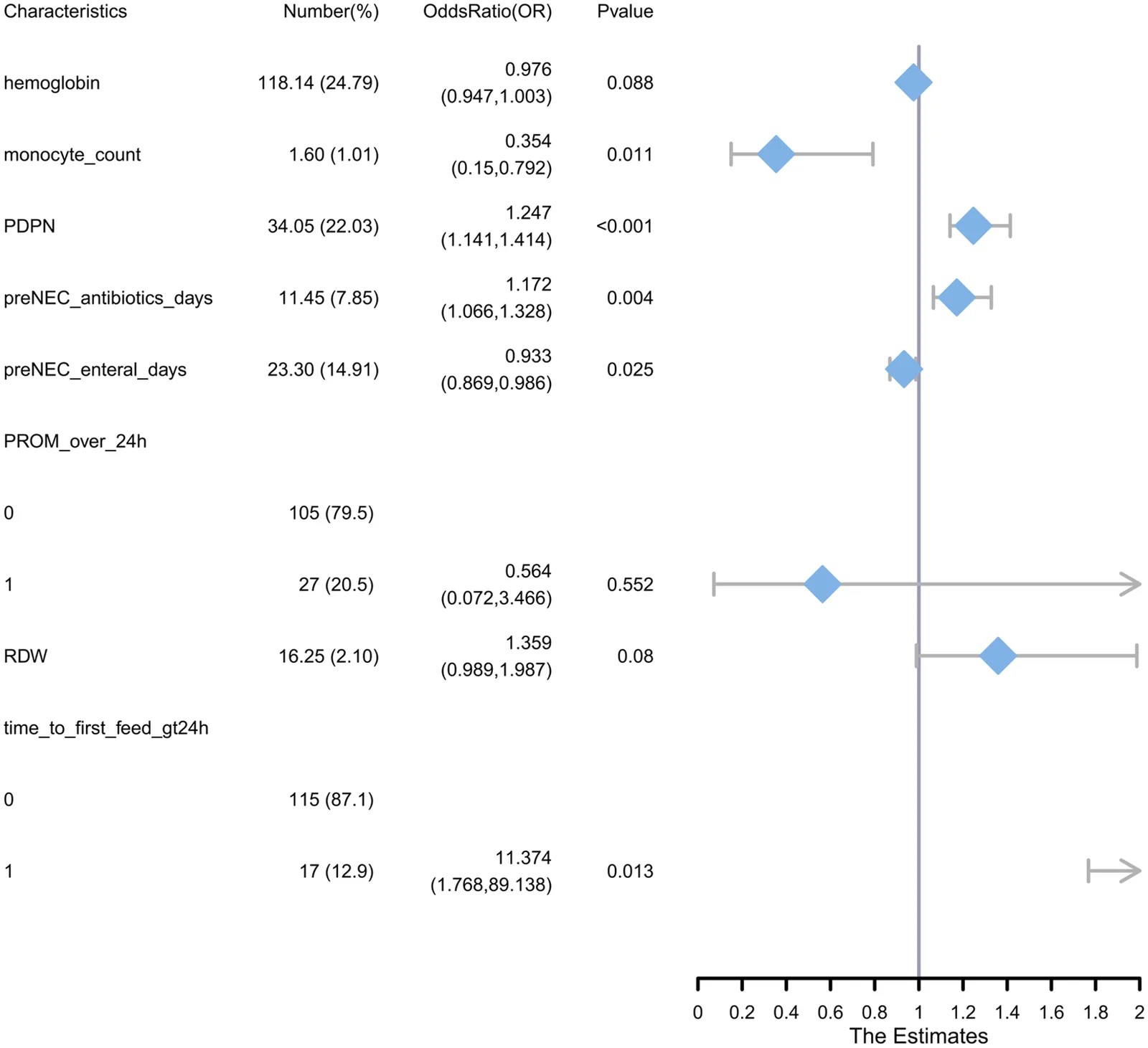
Multivariable logistic regression analysis of LASSO-selected variables for NEC occurrence. Forest plot showing the adjusted odds ratios and 95% confidence intervals of the variables.

### Machine learning model development and comparison

Machine-learning models were developed from clinical variables, laboratory measures, serum PDPN, and derived immunoinflammatory biomarkers obtained before NEC onset. Their internal performance was compared across discrimination, calibration, precision-recall, and clinical utility measures (**Supplementary Table S2**).

CatBoost performed most favorably among the candidate algorithms and was selected for interpretation (**Figure 8**). Receiver operating characteristic and precision-recall analyses indicated strong discrimination, while calibration showed acceptable agreement between predicted and observed risk. Decision curve analysis suggested net benefit across a clinically relevant range of threshold probabilities.

**Figure 8:**
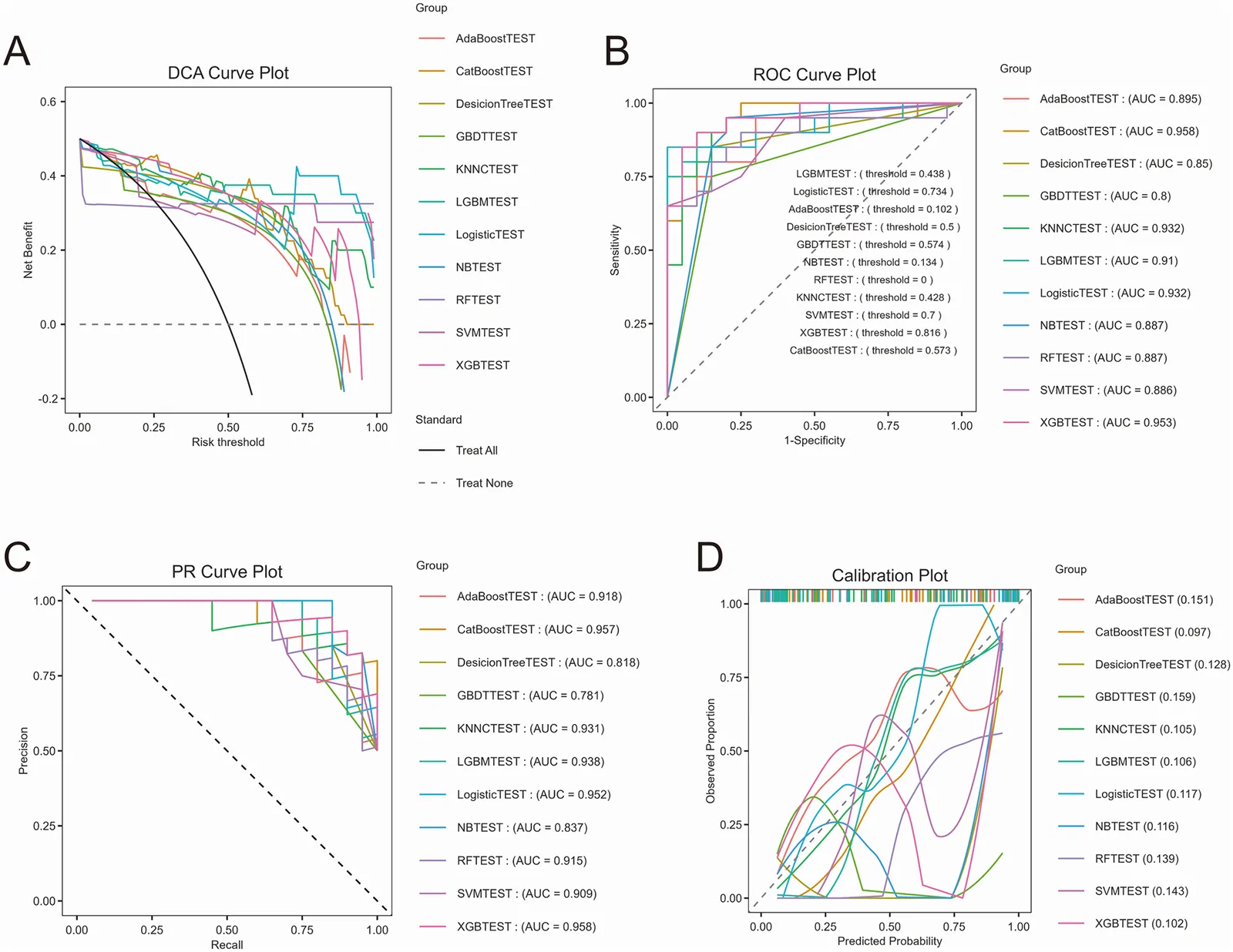
Performance comparison of machine learning models for NEC identification. **(A)** Decision curve analysis. **(B)** Receiver operating characteristic curves. **(C)** Precision-recall curves. **(D)** Calibration plots.

### SHAP-based interpretation of the CatBoost model

SHAP was used to examine the behavior of the CatBoost model. PDPN had the largest mean absolute SHAP value and therefore ranked first in global importance (**Figure 9E**). This result paralleled its elevation in NEC, strong individual discrimination, and independent association in the multivariable logistic model.

**Figure 9:**
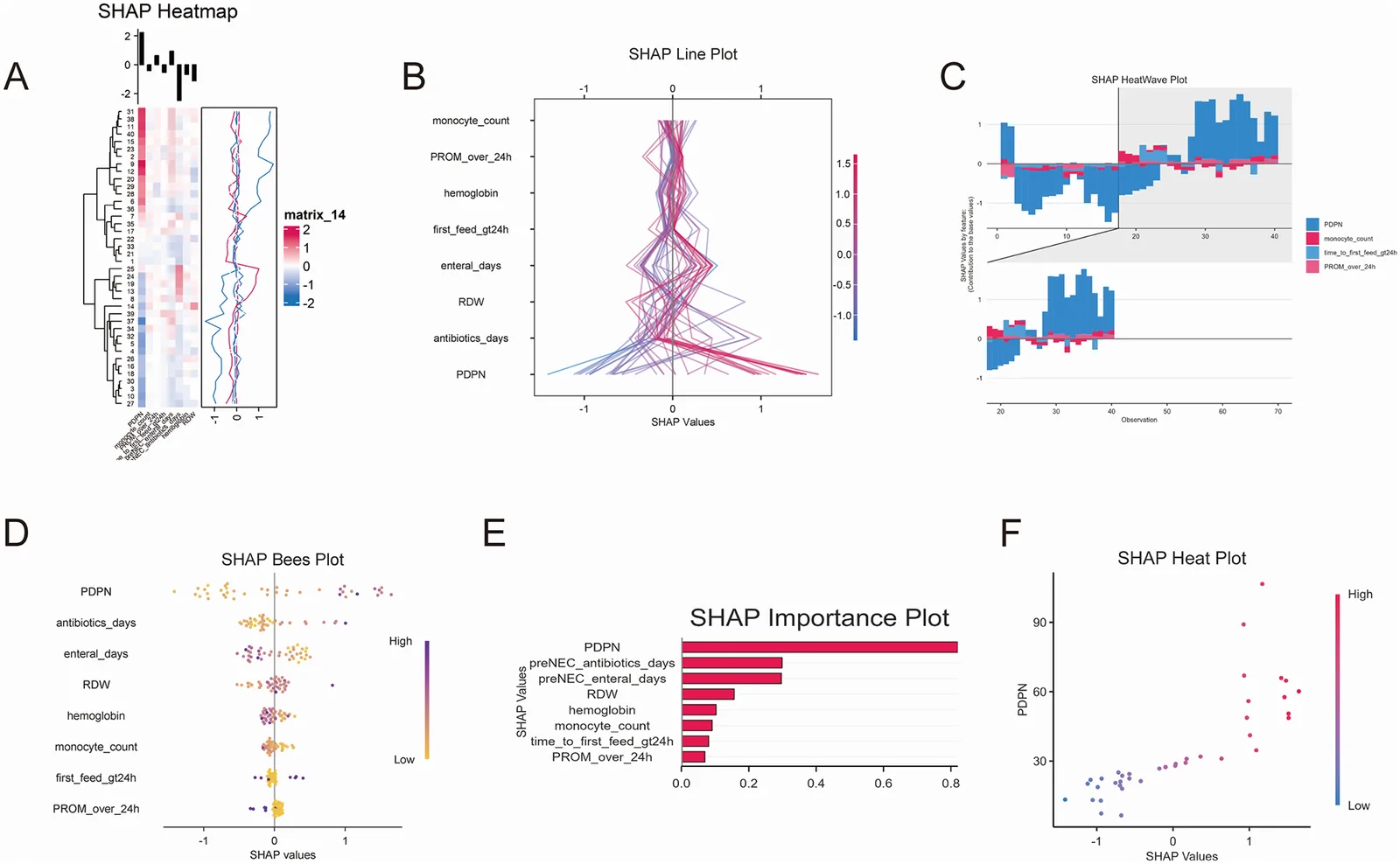
SHAP-based interpretation of the CatBoost model. **(A)** SHAP heatmap. **(B)** SHAP line plot. **(C)** SHAP heatwave plot. **(D)** SHAP beeswarm plot. **(E)** SHAP feature importance plot. **(F)** SHAP dependence plot.

Other contributors included antibiotic and enteral-feeding durations, red cell distribution width, hemoglobin, monocyte count, time to first enteral feeding >24 h, and premature rupture of membranes >24 h. Higher PDPN values generally shifted predictions toward NEC in the beeswarm plot (**Figure 9D**), and the dependence plot showed a positive relation between PDPN concentration and SHAP value (**Figure 9F**). Individual visualizations varied across infants, indicating that no single combination of biomarker, hematologic, feeding, and perinatal features accounted for all predictions (**Figures 9A–C**).

### Nomogram construction and internal assessment

A logistic regression-based nomogram was constructed from the eight LASSO-selected variables to provide a more accessible presentation of the multivariable model (**Figure 10A**). It included serum PDPN, monocyte count, premature rupture of membranes >24 h, time to first enteral feeding >24 h, duration of enteral feeding before NEC, duration of antibiotic use before NEC, hemoglobin, and red cell distribution width.

**Figure 10:**
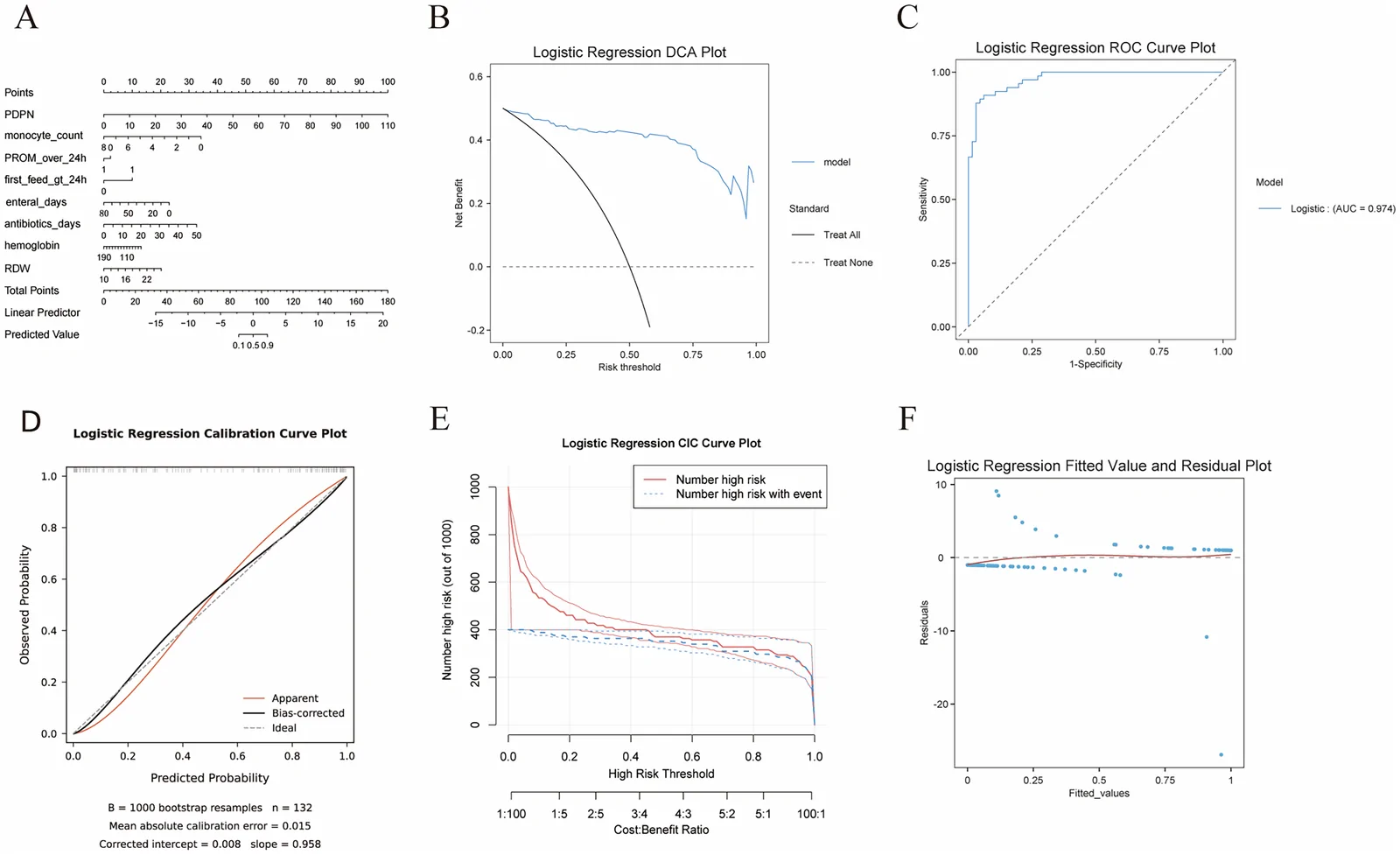
Construction and internal assessment of the logistic regression-based nomogram. **(A)** Nomogram for individualized NEC risk estimation. **(B)** Decision curve analysis. **(C)** Receiver operating characteristic curve. **(D)** Calibration curve. **(E)** Clinical impact curve. **(F)** Fitted value and residual plot.

On internal assessment, the nomogram produced net benefit over the treat-all and treat-none strategies across a clinically relevant threshold range (**Figure 10B**). Its area under the receiver operating characteristic curve was 0.974 (**Figure 10C**). Bootstrap internal validation yielded a mean absolute calibration error of 0.015 and optimism-corrected calibration intercept and slope of 0.008 and 0.958, respectively (**Figure 10D**). The clinical impact curve supported potential usefulness in identifying higher-risk infants (**Figure 10E**). No clear systematic pattern was apparent in the fitted-value and residual plot (**Figure 10F**).

## Discussion

This nested case-control study tested a biologically grounded PDPN hypothesis by linking its expression in public NEC intestinal-tissue datasets to serum measurements obtained before clinical diagnosis. PDPN was upregulated in both transcriptomic datasets, elevated in infants who subsequently developed NEC, and higher in Bell stage III than in stage II disease. Its discrimination exceeded that of the conventional immunoinflammatory indices examined, and the association persisted after multivariable adjustment. CatBoost had the most favorable internal performance among the candidate algorithms; within that model, SHAP ranked PDPN as the leading contributor. The nomogram offered a simpler, regression-based view of the same pre-NEC variables. Taken together, the findings support further evaluation of serum PDPN as part of individualized NEC risk stratification.

Previous clinical studies provide context for the association between circulating PDPN and NEC. Huang and Tang reported that serum PDPN was associated with disease activity and inflammatory and coagulation markers in a cross-sectional study of adults with ulcerative colitis (34). Ji et al. found elevated soluble PDPN levels and associations with markers of hypercoagulability in patients with nephrotic syndrome (35). The present study extends these observations to serum samples obtained before NEC diagnosis in preterm infants. PDPN showed weak positive correlations with high-sensitivity C-reactive protein and CAR, but no significant correlations with the other cell-count-derived indices examined. These findings suggest partial overlap with conventional inflammatory measures and warrant further evaluation of PDPN as a complementary biomarker.

The biological effects of PDPN depend on the disease context. PDPN participates in lymphatic and stromal remodeling, immune-cell trafficking, and platelet signaling (13–18). Rayes et al. demonstrated a protective role for the PDPN–CLEC-2 axis in limiting inflammation and organ injury in mouse models of sepsis (36), while other studies have implicated this pathway in thromboinflammation and epithelial repair (37, 38). These findings provide biological context but do not establish the source or function of elevated circulating PDPN in NEC. Circulating PDPN may reflect inflammatory, tissue-injury, or reparative processes; its mechanistic role in NEC remains unresolved.

Derived immunoinflammatory biomarkers discriminated NEC less well than PDPN. Such modest performance is unsurprising given the changing mixture of inflammation, barrier injury, microbial dysbiosis, feeding intolerance, hypoperfusion, and host immaturity before clinical deterioration (1, 2, 7, 8). Cell-count-derived indices are inexpensive and readily available, but gestational age, postnatal adaptation, infection, transfusion, antibiotic exposure, and other neonatal conditions can alter them (9, 39, 40). Their limited specificity makes them unsuitable as stand-alone indicators in very preterm infants. They may still contribute complementary information when considered alongside PDPN and clinical variables.

A multivariable approach is well suited to NEC because preclinical trajectories differ among infants and are unlikely to be represented by a single biomarker (19, 41). Evidence of interacting microbial, metabolic, and immune pathways in NEC and neonatal lupus further illustrates the complexity of neonatal inflammation (42, 43). In our cohort, CatBoost integrated inflammatory, feeding, antibiotic-exposure, hematologic, and perinatal information and showed the strongest internal performance among the evaluated algorithms. PDPN was its leading SHAP contributor, supporting further investigation of this biomarker within multivariable NEC risk assessment.

The logistic regression-based nomogram was developed because strong algorithmic performance alone does not guarantee clinical usability. At the bedside, a risk tool must communicate its estimates clearly as well as discriminate accurately (44). The nomogram converts the LASSO-selected variables into a visual estimate based on PDPN and routine pre-NEC data. It serves a different purpose from CatBoost: the latter can accommodate nonlinearities and interactions, whereas the nomogram is easier to inspect and discuss. Neither should be considered ready for clinical decision-making without external validation (45).

A strength of this study is that public transcriptomic data were used to examine a biologically prespecified candidate before serum validation in a defined clinical cohort. The same infants contributed PDPN measurements, clinical and laboratory variables, and derived immunoinflammatory indices, maintaining consistency across the clinical analyses. Restricting every predictor to information available before NEC onset aligned model development with early risk assessment rather than *post hoc* classification. Conventional regression, machine learning, SHAP, and the nomogram then provided complementary views of association, prediction, and interpretability.

The findings should be interpreted in light of several limitations. This single-center study had a modest sample size and test set, limiting the precision of performance estimates for the machine-learning models. Internal validation cannot replace evaluation in independent, prospectively enrolled multicenter cohorts. We did not include a separate comparator group of infants with sepsis but without NEC. Future studies incorporating clinically comparable NEC and non-NEC sepsis groups are needed to assess this distinction. Finally, further mechanistic studies are warranted to clarify the role of circulating PDPN in NEC pathogenesis and its relationship with inflammation and tissue injury.

## Conclusions

Serum PDPN is a promising candidate for early NEC risk stratification in preterm infants. Public transcriptomic evidence, prediagnostic serum validation, and complementary statistical and machine-learning analyses consistently linked PDPN to NEC occurrence and Bell stage. PDPN was also the principal contributor to CatBoost predictions. Combining it with routine clinical and laboratory data may enable individualized risk estimation, although the present models require prospective multicenter validation. Mechanistic studies are also needed to define the role of PDPN in NEC pathogenesis.

## Data Availability

The publicly available intestinal transcriptomic datasets analyzed in this study are available in the NCBI Gene Expression Omnibus (GEO) under accession numbers GSE46619 (https://www.ncbi.nlm.nih.gov/geo/query/acc.cgi?acc=GSE46619) and GSE64801 (https://www.ncbi.nlm.nih.gov/geo/query/acc.cgi?acc=GSE64801). The individual-level clinical, laboratory, and serum PDPN data are not publicly available to protect participant privacy. Requests for access to them should be directed to the corresponding authors and will be considered in accordance with the terms of consent, institutional policies, and applicable ethical requirements.
