# Supplementary material for: Transcriptomics-guided serum podoplanin and interpretable machine learning for early risk stratification of necrotizing enterocolitis in preterm infants
Source: Front Immunol. 2026 Oct 6;17:1973760. doi: 10.3389/fimmu.2026.1973760 (PMC13680146; doi:10.3389/fimmu.2026.1973760)
Supplement: Supplementary file 1 [file Table1.docx]

# Supplementary information

**Supplementary Table S1.** Full baseline characteristics of the study cohort.

| **Variables** | **Control (n=66)** | **NEC (n=66)** | ***P*-value** |
| --- | --- | --- | --- |
| Maternal age, years | 31.12 (4.99) | 30.14 (3.36) | 0.187 |
| Assisted reproduction, n (%) | 12.00 (18.18%) | 8.00 (12.12%) | 0.332 |
| Gestational hypertension, n (%) | 19.00 (28.79%) | 15.00 (22.73%) | 0.426 |
| Gestational diabetes mellitus, n (%) | 19.00 (28.79%) | 14.00 (21.21%) | 0.315 |
| Placental abruption, n (%) | 4.00 (6.06%) | 5.00 (7.58%) | 0.730 |
| PROM >24 h, n (%) | 18.00 (27.27%) | 9.00 (13.64%) | 0.052 |
| Chorioamnionitis, n (%) | 1.00 (1.52%) | 1.00 (1.52%) | >0.999 |
| Antenatal magnesium sulfate, n (%) | 20.00 (30.30%) | 24.00 (36.36%) | 0.460 |
| Antenatal antibiotics, n (%) | 19.00 (28.79%) | 14.00 (21.21%) | 0.315 |
| Antenatal steroids, n (%) | 54.00 (81.82%) | 50.00 (75.76%) | 0.394 |
| Cesarean delivery, n (%) | 34.00 (51.52%) | 31.00 (46.97%) | 0.601 |
| Gestational age, weeks | 30.08 (1.94) | 29.72 (2.07) | 0.303 |
| Birth weight, g | 1218.79 (342.94) | 1229.79 (316.54) | 0.848 |
| Male sex, n (%) | 37.00 (56.06%) | 36.00 (54.55%) | 0.861 |
| SGA, n (%) | 20.00 (30.30%) | 19.00 (28.79%) | 0.849 |
| Multiple birth, n (%) | 50.00 (75.76%) | 55.00 (83.33%) | 0.281 |
| Nuchal cord, n (%) | 30.00 (45.45%) | 32.00 (54.24%) | 0.327 |
| Apgar score at 1 min >7, n (%) | 49.00 (77.78%) | 41.00 (68.33%) | 0.237 |
| Apgar score at 5 min <8, n (%) | 5.00 (7.69%) | 6.00 (9.38%) | 0.732 |
| Body temperature, °C | 35.95 (0.51) | 35.94 (0.51) | 0.906 |
| Time to first enteral feeding >24 h, n (%) | 5.00 (7.58%) | 10.00 (15.87%) | 0.142 |
| Initial breast milk feeding, n (%) | 6.00 (9.09%) | 3.00 (4.55%) | 0.300 |
| Duration of enteral feeding before NEC, days | 28.83 (12.95) | 18.18 (14.17) | <0.001 |
| Duration of NPO before NEC, days | 3.12 (4.07) | 4.25 (4.91) | 0.158 |
| Reached full enteral feeding, n (%) | 25.00 (37.88%) | 36.00 (57.14%) | 0.028 |
| Full enteral feeding before NEC, n (%) | 43.00 (65.15%) | 22.00 (34.92%) | <0.001 |
| Pulmonary surfactant use, n (%) | 31.00 (46.97%) | 30.00 (45.45%) | 0.861 |
| Caffeine use before NEC, n (%) | 43.00 (65.15%) | 43.00 (66.15%) | 0.904 |
| Duration of caffeine use before NEC, days | 18.15 (15.89) | 12.81 (13.42) | 0.047 |
| Intravenous fluid use before NEC, n (%) | 31.00 (46.97%) | 29.00 (44.62%) | 0.787 |
| Duration of intravenous fluid use before NEC, days | 6.33 (9.30) | 6.17 (9.65) | 0.926 |
| Inotrope use before NEC, n (%) | 26.00 (39.39%) | 31.00 (47.69%) | 0.338 |
| Duration of inotrope use before NEC, days | 3.58 (7.31) | 3.54 (5.42) | 0.974 |
| Ibuprofen for PDA before NEC, n (%) | 7.00 (10.61%) | 6.00 (9.38%) | 0.815 |
| Duration of ibuprofen use before NEC, days | 0.42 (1.18) | 0.41 (1.21) | 0.963 |
| Steroid use before NEC, n (%) | 11.00 (16.67%) | 9.00 (14.06%) | 0.681 |
| Duration of steroid use before NEC, days | 1.61 (5.33) | 0.76 (2.49) | 0.280 |
| RBC transfusion before NEC, n (%) | 37.00 (56.06%) | 36.00 (55.38%) | 0.938 |
| RBC transfusion before NEC, units | 2.46 (3.39) | 2.24 (3.02) | 0.712 |
| Probiotics use, n (%) | 27.00 (40.91%) | 26.00 (40.63%) | 0.974 |
| Antibiotic use before NEC, n (%) | 60.00 (90.91%) | 64.00 (98.46%) | 0.055 |
| Duration of antibiotic use before NEC, days | 9.23 (4.68) | 13.84 (9.54) | <0.001 |
| UVC use, n (%) | 29.00 (43.94%) | 29.00 (46.03%) | 0.811 |
| Duration of UVC use, days | 3.12 (3.66) | 3.32 (3.50) | 0.769 |
| PICC use before NEC, n (%) | 47.00 (71.21%) | 47.00 (73.44%) | 0.777 |
| Duration of PICC use, days | 18.53 (18.61) | 21.53 (21.18) | 0.415 |
| Respiratory distress syndrome, n (%) | 41.00 (62.12%) | 37.00 (56.06%) | 0.479 |
| PDA, n (%) | 21.00 (31.82%) | 31.00 (48.44%) | 0.053 |
| White blood cell count, ×10⁹/L | 10.95 (4.06) | 9.57 (4.92) | 0.082 |
| Neutrophil count, ×10⁹/L | 4.25 (2.95) | 4.95 (8.54) | 0.536 |
| Lymphocyte count, ×10⁹/L | 4.39 (1.48) | 4.10 (3.29) | 0.511 |
| Monocyte count, ×10⁹/L | 1.75 (0.89) | 1.44 (1.09) | 0.072 |
| Platelet count, ×10⁹/L | 326.09 (126.87) | 250.48 (137.84) | 0.001 |
| Hemoglobin, g/L | 123.14 (22.51) | 113.74 (26.00) | 0.029 |
| Hematocrit | 0.36 (0.07) | 0.34 (0.08) | 0.076 |
| RDW, % | 15.87 (1.89) | 16.62 (2.26) | 0.043 |
| MCV, fL | 101.01 (7.53) | 99.35 (8.34) | 0.232 |
| MPV, fL | 12.75 (11.64) | 11.18 (1.06) | 0.284 |
| PDW, % | 13.82 (3.43) | 13.99 (2.79) | 0.761 |
| PCT, ng/mL | 0.36 (0.12) | 0.30 (0.16) | 0.037 |
| hs-CRP, mg/L | 4.27 (10.37) | 9.66 (17.21) | 0.052 |
| Albumin, g/L | 32.16 (5.41) | 30.77 (3.97) | 0.133 |
| Total bilirubin, μmol/L | 96.68 (53.61) | 87.66 (51.41) | 0.378 |
| ALP, U/L | 273.79 (128.93) | 291.47 (149.21) | 0.491 |
| LDH, U/L | 375.83 (186.76) | 440.49 (211.93) | 0.080 |
| Total bile acids, μmol/L | 7.19 (6.69) | 9.04 (11.32) | 0.289 |
| Serum PDPN, ng/mL | 19.75 (6.26) | 48.36 (22.89) | <0.001 |
| CAR | 0.12 (0.31) | 0.32 (0.73) | 0.053 |
| PLR | 76.93 (28.72) | 71.01 (39.47) | 0.328 |
| NLR | 1.05 (0.81) | 1.15 (0.87) | 0.463 |
| SII | 316.13 (246.13) | 268.09 (243.24) | 0.263 |
| LMR | 3.04 (1.59) | 3.69 (2.36) | 0.070 |
| SIRI | 2.11 (2.49) | 2.02 (3.07) | 0.854 |
| PNR | 110.73 (77.15) | 98.25 (77.33) | 0.357 |
| NMR | 2.51 (1.20) | 5.22 (11.68) | 0.066 |

Values are presented as mean (SD) or n (%). NEC, necrotizing enterocolitis; SGA, small for gestational age; PROM, premature rupture of membranes; NPO, nil per os; RBC, red blood cell; UVC, umbilical venous catheter; PICC, peripherally inserted central catheter; RDS, respiratory distress syndrome; PDA, patent ductus arteriosus; RDW, red cell distribution width; MCV, mean corpuscular volume; MPV, mean platelet volume; PDW, platelet distribution width; PCT, procalcitonin; hs-CRP, high-sensitivity C-reactive protein; CAR, C-reactive protein-to-albumin ratio; PDPN, podoplanin; PLR, platelet-to-lymphocyte ratio; NLR, neutrophil-to-lymphocyte ratio; SII, systemic immune-inflammation index; LMR, lymphocyte-to-monocyte ratio; SIRI, systemic inflammation response index; PNR, platelet-to-neutrophil ratio; NMR, neutrophil-to-monocyte ratio.

**Supplementary Table S2.** Performance metrics of candidate machine learning models for NEC identification.

| **Model** | **Accuracy** | **Sensitivity** | **Specificity** | **Precision** | **F1-score** | **MCC** | **AUROC** | **FNR** | **FPR** |
| --- | --- | --- | --- | --- | --- | --- | --- | --- | --- |
| CatBoost | 0.850 | 0.850 | 0.850 | 0.850 | 0.850 | 0.700 | 0.958 | 0.150 | 0.150 |
| XGBoost | 0.850 | 0.900 | 0.800 | 0.818 | 0.857 | 0.704 | 0.953 | 0.100 | 0.200 |
| SVM | 0.850 | 0.700 | 1.000 | 1.000 | 0.824 | 0.734 | 0.910 | 0.300 | 0.000 |
| KNN | 0.775 | 0.700 | 0.850 | 0.824 | 0.757 | 0.556 | 0.886 | 0.300 | 0.150 |
| Random forest | 0.850 | 0.850 | 0.850 | 0.850 | 0.850 | 0.700 | 0.932 | 0.150 | 0.150 |
| Naive Bayes | 0.825 | 0.650 | 1.000 | 1.000 | 0.788 | 0.694 | 0.887 | 0.350 | 0.000 |
| GBDT | 0.850 | 0.900 | 0.800 | 0.818 | 0.857 | 0.704 | 0.888 | 0.100 | 0.200 |
| Decision tree | 0.800 | 0.750 | 0.850 | 0.833 | 0.789 | 0.603 | 0.800 | 0.250 | 0.150 |
| AdaBoost | 0.850 | 0.850 | 0.850 | 0.850 | 0.850 | 0.700 | 0.850 | 0.150 | 0.150 |
| Logistic regression | 0.800 | 0.800 | 0.800 | 0.800 | 0.800 | 0.600 | 0.895 | 0.200 | 0.200 |
| LightGBM | 0.825 | 0.850 | 0.800 | 0.810 | 0.829 | 0.651 | 0.933 | 0.150 | 0.200 |

MCC, Matthews correlation coefficient; AUROC, area under the receiver operating characteristic curve; FNR, false-negative rate; FPR, false-positive rate; NEC, necrotizing enterocolitis; SVM, support vector machine; KNN, k-nearest neighbors; GBDT, gradient boosting decision tree; LightGBM, light gradient boosting machine.
